# Brighter-colored paper wasps (*Polistes dominula*) have larger poison glands

**DOI:** 10.1186/1742-9994-9-20

**Published:** 2012-08-20

**Authors:** J Manuel Vidal-Cordero, Gregorio Moreno-Rueda, Antonio López-Orta, Carlos Marfil-Daza, José L Ros-Santaella, F Javier Ortiz-Sánchez

**Affiliations:** 1 Departamento de Zoología, Facultad de Ciencias, Universidad de Granada, E-18071, Granada, Spain; 2 Grupo de Investigación “Transferencia de I + D en el Área de Recursos Naturales”, Universidad de Almería, ctra. de Sacramento, s/n, E-04120, La Cañada de San Urbano, Almería, Spain

**Keywords:** Paper wasp, Poison glands, Aposematic coloration, Warning signals

## Abstract

**Introduction:**

Aposematism is a defense system against predators consisting of the toxicity warning using conspicuous coloration. If the toxin production and aposematic coloration is costly, only individuals in good physical condition could simultaneously produce abundant poison and striking coloration. In such cases, the aposematic coloration not only indicates that the animal is toxic, but also the toxicity level of individuals. The costs associated with the production of aposematic coloration would ensure that individuals honestly indicate their toxicity levels. In the present study, we examine the hypothesis that a positive correlation exists between the brightness of warning coloration and toxicity level using as a model the paper wasp (*Polistes dominula*).

**Results:**

We collected wasps from 30 different nests and photographed them to measure the brightness of warning coloration in the abdomen. We also measured the volume of the poison gland, as well as the length, and the width of the abdomen. The results show a positive relationship between brightness and poison-gland size, which remained positive even after controlling for the body size and abdomen width.

**Conclusion:**

The results suggest that the coloration pattern of these wasps is a true sign of toxicity level: wasps with brighter colors are more poisonous (they have larger poison glands).

## Introduction

Aposematic coloration is a defense system against predators widely used in the animal kingdom, by which potential prey use their striking coloration to warn a possible predator that they are toxic [1,2]. Aposematism has learning components, because predators learn to associate aposematic coloration with toxicity after testing the prey [3]. Thus, predators learn to avoid distasteful prey more quickly when the prey is more visible, as opposed to cryptic prey [4], which, in turn, impels a selective pressure for toxic prey to be as striking as possible, leading to a coevolution between predator and prey [5].

Becoming and remaining toxic is likely to be costly for individuals, due to costs associated with the production or storage of the toxin [6]. In this case, once predators have learned to avoid aposematic prey coloration, an individual would benefit from having aposematic coloration and decrease the demands of toxicity [7]. This would cause the evolution of warning coloration to be evolutionarily unstable. In this situation, individuals within a population will honestly warn of their toxicity using warning coloration only if the signal is difficult to produce, so that a cheater would not be able to cope with such demands [8]. For example, since the warning coloration is striking, it would attract inexperienced predators, so that an individual with non-toxic aposematic coloration would be attacked with greater probability than an individual with cryptic coloration [9]. In such a case, the cost of attracting predators may be tolerated only by animals which are the brightest and the most toxic in the population [10]. Aposematic color production can also be metabolically costly [11]. Additionally, certain pigment molecules used in the aposematic coloring (e.g., carotenoids, melanin) can act as antioxidants [12,13]. Consequently, investing in a brighter coloration would be demanding for the animal, making it more susceptible to oxidative stress, because the pigments used in coloration are diverted from combating oxidative stress [14].

Therefore, if the toxin production and aposematic coloration is demanding, only individuals in good physical condition can simultaneously sustain the production of abundant poison and striking coloration [15]. Therefore aposematic coloration would be a true indicator of the toxicity level in individuals [14]. In fact, birds are able to distinguish subtle differences in the coloration of prey, and they are more cautious with the most colorful individuals [16]. Supporting this idea, ladybirds (*Harmonia axyridis* and *Coccinella septempunctata*) show a positive correlation between brightness and toxicity [15,17]. Although there is evidence of intraspecific variation in levels of aposematic coloration [18], there are few studies that relate the characteristics of the coloring, such as the brightness, with the poison amount [15,17].

In the present study we tested the hypothesis that there is a positive correlation between the brightness of the warning coloration and toxicity using the paper wasp (*Polistes dominula*) as a study model. These wasps, in order to avoid predators, have conspicuous color patterns in yellow and black covering their bodies [19], and stings armed with the poison gland. The coloration of these wasps depends on environmental conditions [20] but no study is available on the relationship between the brightness of the coloration and the poison levels. If color is a honest sign of toxicity, wasps with most intense colors (brighter) should be equipped with larger poison glands (and therefore should be able to inject more venom into a potential enemy). To test this prediction, we analyze the relationship between brightness and size of the poison glands in this species.

## Results

**Figure 1:**
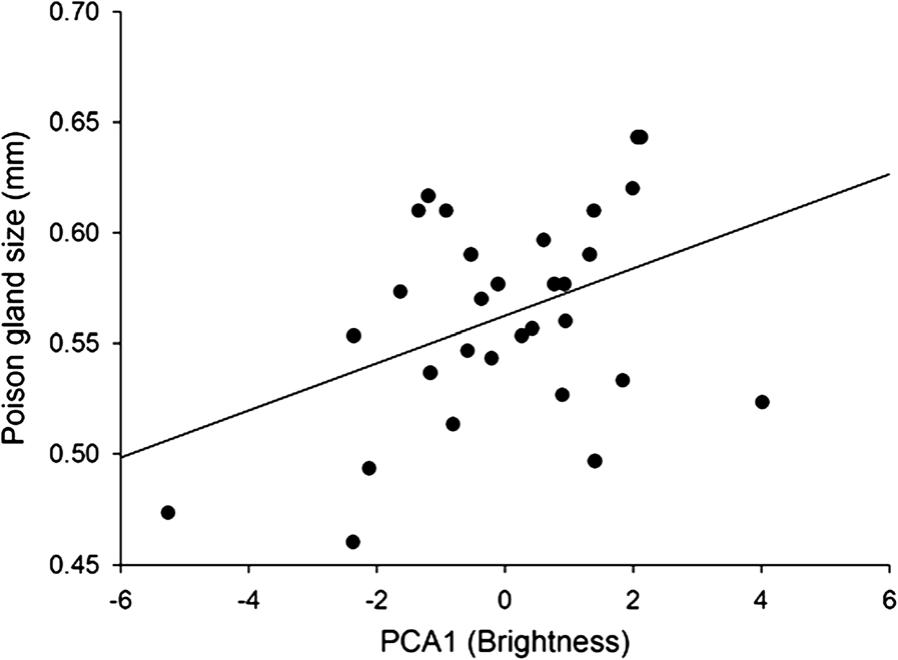
**Relationship between PCA1 factor (brightness) and poison-gland diameter. **The line indicates the regression slope.

The individuals collected showed a poison gland with an average diameter of 0.56 ± 0.01 mm (mean ± SE). The size of the poison gland was not correlated with overall body size of the insect (PCA2, r = 0.15, p = 0.43). However, we found a positive correlation between abdomen width and the size of the poison gland (r = 0.37, p = 0.047). The length of the abdomen and head width did not correlate with the poison-gland size (r = 0.26, p = 0.17, and r = 0.25, p = 0.18, respectively). In terms of color, we found a significant correlation between PCA1 factor (indicator of the overall brightness) and the size of the poison gland (r = 0.41, p = 0.025, Figure 1). This correlation indicates that individuals with larger poison glands had a brighter body coloration. The relationship between brightness and size of the poison gland remained statistically significant after controlling for body size and the width of the abdomen (Table 1).

**Table 1:** **Multiple-regression model relating poison-gland size with coloration (PCA1 factor), controlling for general body size (PCA2 factor; F**_**2, 27**_ **= 3.15, p = 0.059, R**^**2**^ **= 0.19) and for abdomen width (F**_**2, 27**_ **= 4.61, p = 0.019, R**^**2**^ **= 0.25)**

**Effect**	**Β**	**F**_**1, 27**_	**P**
Controlling for general body size (PCA2)			
PCA1	0.41	5.57	0.026
PCA2	0.15	0.73	0.40
Controlling for abdomen width			
PCA1	0.35	4.39	0.046
Abdomen width	0.30	3.16	0.087

## Discussion

The results of our study show that the size of the poison glands in the paper wasp is positively correlated with the brightness of aposematic coloration of the dorsum of the abdomen. This relationship was not confounded by the size of the insect. Although wasps with wider abdomens showed larger glands, when we controlled for the size of the abdomen, the relationship between the size of the poison gland and the color remained statistically significant. Therefore, the wasps with intense coloration probably have more venom to inject, making them more dangerous for predators. Consequently, our results support the idea that paper wasps indicate their level of toxicity through color, a result similar to that found previously in ladybirds [15,17]. Individuals with brighter colors, indicating more poison, can be more easily detected by a predator. Therefore, the predator can assess the risk involved in attacking the potential prey and can decide not to attack if the prey is very dangerous [10,16]. In the case of novice predators, although brighter coloration would seem more attractive, the signal may be associated with more toxic animals, making the defense successful. There is evidence that aposematic prey can survive attacks by inexperienced predators [9]. Moreover, given that these wasps are eusocial, by attacking a very poisonous wasp, the predator may easily learn to avoid the remaining individuals in the colony. Our results therefore support theoretical models that predict that warning coloration can indicate the level of toxicity, not only that the animal is toxic [10,14].

The positive correlation we found between poison gland size and color suggests that only individuals in good physical condition can cope with the process of developing a significant amount of poison while maintaining an intense color (see [15]). This would occur if the production of the coloration and the venom compete for a given resource. It can be assumed that both processes require ample energy. Another possibility is that poison production is demanding in terms of oxidative stress, and that the pigments used in aposematic coloring have an antioxidant function [14]. In both cases, individuals would have to reach an optimal level of investment in poison and color according to their body condition, resulting in the observed positive correlation (individuals in better condition being brighter and having larger poison glands). The exact mechanism by which aposematic coloration can indicate the level of toxicity is still unknown, and in fact, we are beginning to discover that color may indicate quantitatively the level of toxicity. An example which shows that aposematic coloration, like other traits, is demanding and requires a trade-off with other aspects of life strategies is shown from the experiments with the wood tiger moth (*Parasemia plantaginis*), in which there is a polymorphism that remains the same as a result of the balance between survival and mating success. In this species, the white morphotype (not aposematic) has more reproductive success, while the yellow morphotype (aposematic) is more successful in survival against predators [21].

The results at the intraspecific level (a positive correlation in three studies, see [15,17]) differ from results at the interspecific level. In frogs of the genus *Epipedobates*, toxicity negatively correlates with warning coloration [22], while other studies have correlated brightness and toxicity in frogs from the dendrobatid family [23] and marine opisthobranchs [24]. At the interpopulation level, no relationship was found between toxicity and coloration between different populations of the frog *Dendrobates pumilio* in a study [25], while a positive correlation was found in a more recent study [26]. These conflicting results could be explained by the variation in the costs and benefits of coloration and toxicity according to the ecological characteristics of species or populations [27]. However, while the model of Speed & Ruxton [27] applies to interpopulation or interspecific variation in aposematic coloration and toxicity, predicting positive or negative correlations according to ecological circumstances, the variation within a population can best be explained by the model of Blount et al. [14] (also see [10,28]), which predicts a positive correlation between toxicity and coloration within a given population. This model predicts that warning coloration includes a cost in the signal's production that allows it to be a true indicator of the level of toxicity.

## Conclusion

In conclusion, according to the results of our study, paper wasps appear to indicate their toxicity level by the abdomen color (brighter-colored wasps having larger poison glands). These results imply that aposematic coloration may have evolved as a Zahavian signal, and coloration is an accurate indicator of toxicity. Predators, therefore, can use the information provided by the color of their potential prey to decide whether or not to attack, or to measure the level of caution that they must take in an attack.

## Methods

### The species

*Polistes dominula* has been a good candidate for studies linking the color with other variables, such as the establishment of social status [28-30]. These wasps are eusocial insects, and in southern Spain, the colony-founding process is relatively long (late February to mid-May). The colony process can start with several females, and although all the founders are potentially capable of reproduction, an individual ultimately exerts dominance (alpha queen), laying most of the eggs while the subordinates are responsible for foraging, feeding larvae, and collecting materials for nest building [31-33].

### Measuring the color and morphology

**Figure 2:**
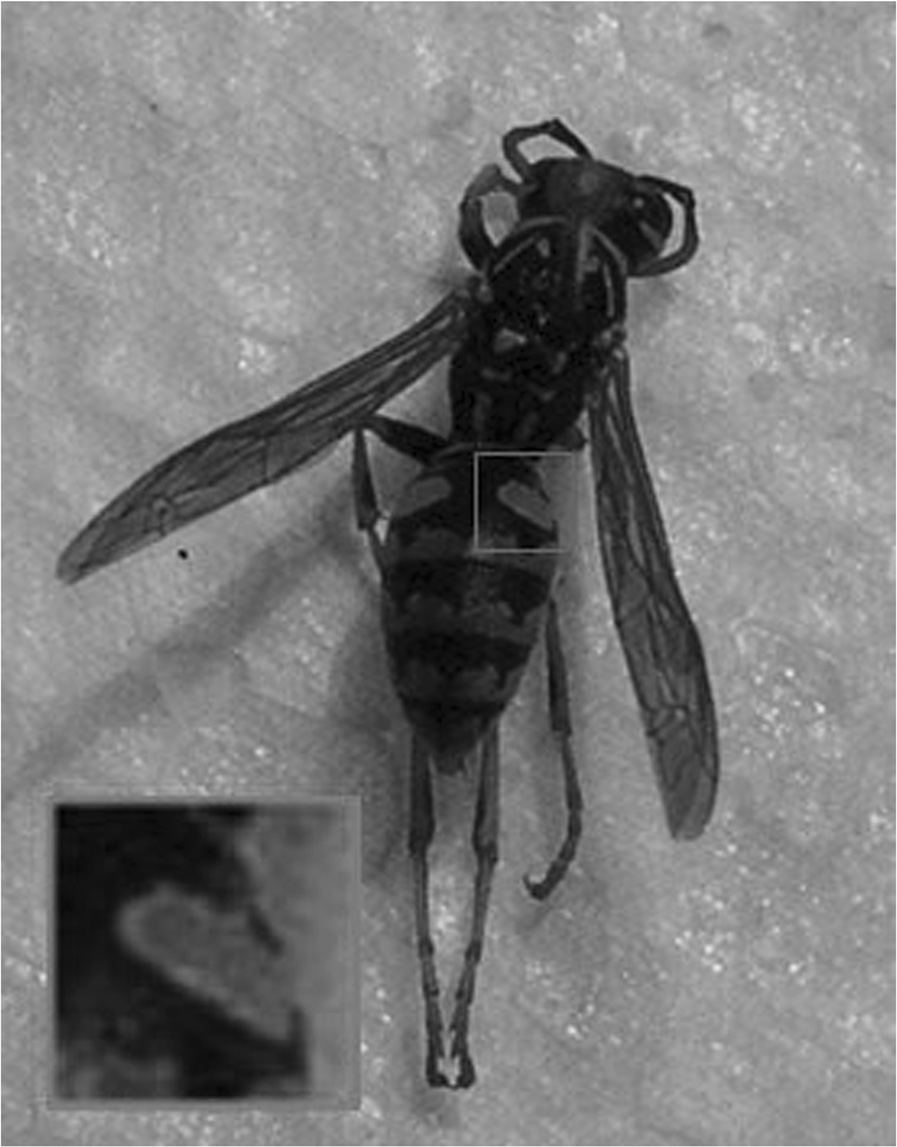
Photograph of the abdomen region of the wasp where the coloration values were taken.

We collected first-year working wasps (we avoided queens and males) from 30 nests in the town of Moraleda de Zafayona (SE Spain). However, note that the exact age of wasps was unknown, and therefore we do not know the possible effect of age on poison gland and coloration. In order to avoid pseudo-replication [34], only one wasp was used per nest. All wasps were collected within 1 hour, and were submerged in 96% ethanol for preservation. One week later, wasps were photographed with a Nikon Coolpix 4.3-megapixel camera. The photographs were taken within 2 hours, under standardized conditions, keeping the camera fixed on a tripod and consistently under the same lighting conditions and background ([35], see e.g. [17]). This made the use of standard gray cards unnecessary. Although ethanol might alter body coloration, all wasps were maintained in ethanol for the same time, and thus alteration would similarly affect every specimen. These photographs were measured for coloration of 10 pixels selected randomly on the right side of yellow band of the second abdominal tergite, as well as 10 other pixels in the black part that divides the second yellow band into two halves (Figure 2). To measure the color variation, we used the program CorelDraw. Coloration was measured with the RGB system [36]. This system gives the color a rating between 0 and 255 for red, green, and blue channels. As in other color-measurement systems, the exact color can be represented by combining the three color coordinates. The higher the value for each channel, the greater the luminance (brightness) for that channel; for example, the coordinate 0, 0, 0 indicates black and 255, 255, 255 indicates white. Using the photographs, we also measured the length and width of the wasp abdomen, and the head width, using the program Image J [37]. Subsequently, the poison gland was removed from the wasp, and its diameter was measured three times, using the average of these three measurements in the analyses. For each case, all measurements were taken by the same researcher.

### Statistical analysis

All variables were normally distributed according to a Shapiro-Wilk's test. Since the variables of color and morphology were correlated among themselves, we reduced the number of predictor variables using principal component analysis (PCA, [38]). The first factor (PCA1) defined color brightness of the insects, as it loaded positively with the value of all the color parameters measured (Table 2). High values of PCA1 indicate that the colors were brighter. The second factor (PCA2) defined body size (Table 2). Higher PCA2 values indicate larger animals. Then the relationship between coloration and body size with the size of the poison gland was examined using Pearson correlations, and the independent effect of each variable was estimated by using multiple regressions. The residuals of the multiple-regression models followed a normal distribution according to a Shapiro-Wilk's test.

**Table 2:** Results of the PCA

**Variable**	**PCA1**	**PCA2**
Red channel (Yellow part)	0.772727	0.211890
Green channel (Yellow part)	0.814046	0.192539
Blue channel (Yellow part)	0.555765	0.191997
Red channel (Black part)	0.673897	-0.439375
Green channel (Black part)	0.705118	-0.537537
Blue channel (Black part)	0.721660	-0.408761
Abdomen length (mm)	0.256526	0.565074
Abdomen width (mm)	0.182501	0.763811
Head width (mm)	0.349572	0.739708

Relationship (loading) among variables of coloration and morphology with the factors PCA1 and PCA2 extracted of the PCA. The two factors explained the 60.9% of variance in coloration and body size.

## Competing interests

The authors declare that they have no competing interests.

## Authors' contributions

GMR conceived of the study. GMR, CMD and JLRS collected specimens in the field. FJOS identified the species. JMVC, CMD and JLRS measured the poison gland. GMR measured the coloration. ALO measured external morphology. GMR performed the statistical analyses. JMVC and GMR wrote the paper with input from the remaining authors. All authors read and approved the final manuscript.
